# A stochastic programming model for trauma hospital network expansion considering rural communities and COVID-19

**DOI:** 10.1007/s10729-025-09719-5

**Published:** 2025-10-04

**Authors:** Eduardo Pérez, Alakshendra Joshi, Sabhasachi Saha, Francis A. Méndez-Mediavilla

**Affiliations:** 1 Ingram School of Engineering, Texas State University, 601 University Drive, 78666 San Marcos, TX USA; 2 Department of Information Systems and Analytics, Texas State University, 601 University Drive, 78666 San Marcos, TX USA

**Keywords:** Trauma, Optimization, Patients, Facility location, Stochastic programming, Population coverage, COVID-19, Operations research, Operations management

## Abstract

Trauma care services are a vital part of all healthcare-based networks as timely accessibility is important for citizens. Trauma care access is even more relevant when unexpected events, such as the COVID-19 pandemic, overload the capacity of the hospitals. Research literature has highlighted that access to trauma care is not even for all populations, especially when comparing rural and urban groups. Traditionally, the focus in trauma systems was on the designation and verification of individual hospitals as trauma centers, rather than on the overall configuration of the system. Recognition of the benefits of an inclusive trauma system has precipitated a more integrated approach. The optimal geographic configuration of trauma care centers is key to maximizing accessibility while promoting the efficient use of resources. This research reports on the development of a two-stage stochastic optimization model for geospatial expansion of a trauma network in a delimited area. The stochastic optimization model recommends the siting of new trauma care centers according to the geographic distribution of the injured population. The model has the potential to benefit both patients and institutions, by facilitating prompt access and promoting the efficient use of resources. The findings indicate that the model significantly improves trauma care coverage, particularly in rural counties, thereby enhancing equitable access to critical healthcare services.

## Highlights

- Trauma care access is crucial, particularly during unexpected events like the COVID-19 pandemic, which can strain hospital capacity.
- Historically, trauma systems focused on individual hospital designation, but recent efforts emphasize a more integrated, inclusive approach.
- A two-stage stochastic optimization model was developed to optimize the geographic distribution of trauma care centers, based on population injury data.
- The model aims to enhance patient access and improve resource efficiency within the trauma care system.

## Introduction

A physical trauma is a body wound occurred due to a physical injury from impact, violence, or accident [1, 2]. Trauma injuries can lead to the death if proper care is not administered to the patient on a timely manner. The importance of designing a good trauma hospital network lies in its ability to save lives, optimize resource utilization, enhance emergency preparedness, and ensure equitable access to high-quality care. By focusing on these aspects, healthcare systems can significantly improve outcomes for trauma patients and bolster overall public health infrastructure.

Equitable access to trauma care centers (TCC) is even more important when considering unexpected events such as the COVID-19 pandemic. TCCs were uniquely impacted by COVID-19 given the need for rapid invasive interventions in severely injured and the growing incidence of community infection [3]. Trauma incidents are one of the leading causes for disability, mortality, and morbidity for patients under the age of 44 in the U.S. and has an economic burden of $671 billion annually [4]. In addition, multiple studies have concluded that access to TCCs is not even for all populations, especially rural and urban groups [5]. Therefore, trauma is a serious health problem with high social and economic costs.

Providing appropriate care to patients suffering trauma injuries requires smooth healthcare delivery processes. Soon after a trauma injury occurs, healthcare paramedics are dispatched to the scene. The paramedics provide first aid to stabilize the patient and then the patient is transported to a TCC. Delays in patient transportation to a trauma center can impact the patient’s survival rate. Clinical intervention is expected within an hour from the moment of an injury incident as a general rule of thumb to avoid negative healthcare outcome [6, 7]. A TCC is a hospital that possesses staff, resources, and equipment needed to provide care to severely injured patients [8]. In the U.S., TCCs are classified by levels according to the type of service they provide [9]. Level-I and Level-II TCCs provide comprehensive services and are equipped to deliver definitive care for patients with severe injuries. In contrast, Level-III and lower-level centers primarily manage non-severe injuries and function as intermediate facilities for stabilizing and transferring patients with more critical conditions.

The goal of this research is to derive new models for the design and/or expansion of a trauma care system within a specified area to improve coverage, specifically in rural areas. Accessing quality trauma care is particularly challenging for rural communities. ZIP codes influence outcomes and rural trauma patients face two major obstacles: distance and time [10]. The optimal geographic configuration of TCCs is key to maximizing accessibility while promoting the efficient use of resources. The aim of this paper is to report on the development of a two-stage stochastic optimization model for geospatial expansion of a trauma network in the state of Texas considering the COVID-19 pandemic. The stochastic programming model recommends the siting of new TCCs by level considering the uncertainty in the geographic distribution of the injured population. Previous efforts to produce data-driven solutions to trauma system design have shortcomings, like assuming a deterministic demand and that every trauma facility serves all types of injuries, prompting the development of a novel approach. The model presented in this paper proves a systematic approach to trauma system design that can help stakeholders in their decisions.

The article proceeds as follows. Section 2 presents a literature review that highlights closely related research. Section 3 presents the problem description, and the parameters associated with the former. Section 4 describes the stochastic programming model. Section 5 presents a case study and the design of experiments to evaluate the feasibility of the current trauma network. Section 6 presents a computational study. Section 7 provides the conclusions reached for the study and Section 8 provides future research prospects and recommendations.

## Literature review

In this section, a review of relevant literature on healthcare facility location and trauma hospital location is provided. Numerous strategies have been proposed to tackle various healthcare facility location challenges. Comprehensive reviews for various healthcare facility location have been conducted by multiple authors including Ahmadi-Javid et al. [8], Reuter-Oppermann et al. [11], and Günes et al. [12]. Next we discuss the works that are more closely related to our research.

### Healthcare facility location in rural areas

Banerji and Fisher [13] dealt with healthcare facility location in rural India. They propose a hierarchical model which considers multiple types of facilities. Berghmans et al. [14] dealt with location of healthcare facilities in a new city. The authors propose a quantitative based method classifying the functionalities of all facilities to be similar rather than hierarchical. Cho [15] proposed a multi-objective model seeking the location of medical service centers and the availability of those services. The model aimed to provide comprehensive healthcare services to patients in an efficient manner pertaining to the quality of the services. The paper defines the measures such as consumer welfare, producer welfare, and the opportunity to obtain service. Rahman et al. [16] proposed a quantitative healthcare facility location-allocation models for developing nations. The authors presented a case study for Guatemala where the *p*-median method was employed taking into consideration population centers and hospital facilities. Mitropoulos et al. [17] proposed a bi-objective model to optimize the location of hospitals and health centers in Greece. The model aimed to minimize the distance between the patient and hospitals and locate adequate facilities to account for multiple population groups and demographics. Hosseini and Ameli [18] implemented the *p*-center method that minimized the maximum distance for all users. The authors expressed that their *p*-median model does not account for emergency services which are needed to operate in rural communities.

Syam and Cote [19] studied the problem of improving the efficiency and effectiveness of the Department of Veterans Affairs (VA) in the United States. They considered various costs incurred by veterans, including family expenditures, and the quality of service provided to veterans. The authors formulated a deterministic optimization model to address the problem. Based on their results, a decentralized system is recommended. Although it is shown that a decentralized system is more expensive, it offers greater access to the population. Kim et al. [20] developed a Lagrange heuristic algorithm to solve the healthcare facility location problem. In their work, they divided patients into groups based on income statistics. Low-income groups were restricted to only access public facilities while medium and high-income groups could access public and private facilities. The model assumed that no new facilities would be added; hence utilizing, to the maximum extent, the use of the existing framework. Their model accomplished the goal of maximizing the number of patients served. Hashmi et al. [21] studied the relation between access to trauma centers and mortality in population groups in the United States. Their results showed that states with poor access to trauma centers have higher mortality rates at the before hospital admission stage of the process. Although their study did not account for scenarios to reduce mortality rates before trauma care, the authors propose the development of a comprehensive database that can track patient outcomes from injury to post discharge to lower mortality outcome statistics.

### Trauma facility location considering patient coverage and access

One of the earliest papers discussing the trauma facility location problem is Branas et al. [7]. The authors proposed an optimization model named the Trauma Resource Allocation Model for Ambulances and Hospitals (TRAMAH). The TRAMAH model was employed to determine the time it takes for a population group to access a trauma center either by ground or aero medical services. Results showed that only 69.2% of all residents had access to either a Level-I or II trauma center within a travel time of 45 min. The model does not consider Level-III and Level-IV trauma centers. In addition, the study mentioned some limitations such as assuming the problem is deterministic which does not address the uncertainty in patient demand for services.

Jansen et al. [22] proposes an algorithm to improve trauma system configuration utilizing existing network and facility locations. The authors used travel times for both ground and air-based services to every hospital in Scotland. Multi-objective performance measurements were considered to account for conflicting objectives of minimizing the total access time and the number of exceptions. Wang et al. [23] proposed the use of an evolutionary algorithm to optimize the problem formulated in Jansen et al. [22]. They validated their approach using real data from Colorado’s trauma system in Jansen et al. [24]. Although the model considered Level-III and below TCCs, it did not account for non-severely injured patients and various destination determination criteria. Brown et al. [25] suggested that unregulated growth of new trauma centers within an existing framework could lead to unforeseen consequences and in their paper evaluated trauma center accessibility with injury mortality across the United States. The authors compiled data from different sources including the Center for Disease Control (CDC) relating to injuries, location of trauma centers from the University of Pennsylvania Cartographic Modeling Laboratory Maps, and the American Trauma Society Trauma Information Exchange Program. The study evaluated the distribution of trauma centers in each state. A Nearest Neighbor ratio (NNR) was devised indicating if the trauma system is clustered or dispersed. The authors calculated NNR as the mean distance between each center and its nearest neighbor while considering the service distributions to be random in each state. The results indicated that the distribution of trauma centers correlates with mortality pertaining to injuries. Clustered trauma centers were associated with lower fatality rates. Reasons for this phenomenon could be better access to these centers in large population regions, yet the authors state that further research is necessary and mention the benefits of using geospatial mapping to plan for new trauma centers.

Horst et al. [26] proposed an approach to add new trauma centers to an existing framework using geospatial mapping. The authors used mapping techniques to layer in data from multiple data systems, from the state of Pennsylvania, such as Pennsylvania Trauma Outcome Study (PTOS), Pennsylvania Trauma Systems Foundation (PTSF), Trauma Mortality Predication Model (TMPM). Road networks were used in calculating the travel times in various zip codes. The study identified 38 trauma centers ranging from Level-I to IV within the PTSF database. Carr et al. [10] also analyzed existing gaps in the Trauma System in the United States using geographic analysis and population estimates. The Trauma Information Exchange Program (TIEP) and the American Trauma Society (ATS) databases were used to identify the trauma system limitations. Geographic data, population demographic data, and access figures (using 60 min as travel time baseline) were considered. The results showed that 88.3% of the population has access to a higher-level trauma center (i.e., Level-I and II), while 11.7% did not.

Gomez et al. [27] developed a model to ensure access to trauma care in the state of New South Wales, Australia. The study used the classification criteria based on the American College of Surgeons (i.e., Level-I to IV) for designating trauma centers. ArcGIS, a geographical information system tool developed by ESRI, was utilized to map locations and analyze transportation networks. Like most studies performed pertaining to trauma care, rotary wing ambulances were used to supplement the travel times between different trauma center designations. The study found that, according to the 2016 Australian Census, 86.1% of the population of New South Wales lives within 60 min to the nearest either Regional Trauma Center or Major trauma Center. The study also concluded that when considering transportation using aircraft and ground-based ambulances, over a 90-minute time, the population able to receive trauma care surges to 99.5%.

### Trauma facility location considering patient mistriages

Mistriage has been utilized in trauma literature as an indicator of patient safety, as it often increases the risk of short and long term disability due to delays in providing definitive care [22, 28–32]. Parikh et al. [28] introduced a model for Performance-based Assessment of Trauma System (PBATS) aimed at identifying the optimal number and locations of Major Trauma Centers (MTCs) while maintaining system-related under-triage (srUT) and over-triage (srOT) rates within predefined limits. Hirpara et al. [29] developed a bi-objective model for the trauma center location problem (TCLP) to determine the number and locations of MTCs and Non-Trauma Centers (NTCs) with the goal of minimizing the weighted sum of srUT and srOT rates. Their approach was demonstrated through a case study based on the existing network of a US state, focusing on both ‘greenfield’ design and the redistribution of existing MTCs. Despite considering both types of patients and associated mistriages, these studies did not explicitly address Intermediate Trauma Centers (ITCs) and various destination determination criteria that impact mistriages.

Hirpara et al. [30] proposed the Nested Trauma Network Design Problem (NTNDP), characterized as a nested multi-level, multi-customer, multi-transportation, multi-criteria, capacitated model with the bi-objective of maximizing the weighted sum of equity and effectiveness in patient safety. Mistriages, including system-related under- and over-triages, were used as surrogates for patient safety. To enhance realism, ITCs were included as feeder centers to major trauma centers, along with three criteria to mimic EMS’s on-scene decisions. A ‘3-phase’ solution approach was proposed, involving solving a relaxed version of the model, a Constraint Satisfaction Problem, and a modified version of the original optimization problem using a commercial solver. Findings indicated that solutions were sensitive to the proportion of assignments attributed to various destination determination criteria, the distribution of trauma patients, and the relative emphasis on equity versus effectiveness. Hirpara et al. [31] introduced a stochastic nested multi-level, multi-transportation capacitated facility location model, explicitly considering mistriages in injury assessment to maximize patient safety by determining the number and locations of MTCs. Using a Bernoulli random variable for moderate and severe injury groups, they proposed a Simheuristic approach integrating Monte Carlo Simulation with a genetic algorithm to convert infeasible solutions into feasible ones and maintain a superior population pool, balancing quality against computational time.

Our review of the literature reveals several unexplored areas in trauma network expansion research. To date, only non-dynamic models have been utilized to address the geographic configuration of TCCs, and these models do not account for uncertainty characteristics. Most models base their recommendations on annual demand, typically for a single year, and assume deterministic demand. Additionally, current models fail to consider the impact of unexpected events such as pandemics like COVID-19. Furthermore, there is a lack of case studies focusing on rural areas. These gaps underscore the need for more comprehensive and dynamic approaches that incorporate uncertainty and address the unique challenges posed by rural settings and unforeseen events.

## Problem description

The problem of maximizing access to TCCs for a population group must consider the location of the population group itself and the location of TCCs that aim to provide services to the group concerned. This study focuses on adult patients aged 15 and older, and TCCs that serve adults. Population groups in this study are identified based on their zip code locations within the specified geographical region. This approach ensures consistency with the demand data provided by the Texas Department of State Health Services (DSHS), which is reported at the zip code level. As is known, the population for any given location, in this case a zip code, is spread across internal communities and zones. Due to this factor, allocating population data to a model is difficult especially when determining which locations can be covered by services. In this paper, a population centroid is used as the geographical coordinate that represents the entire population of said location. This study utilizes population centroids for zip codes that are subsequently used to determine the distances from zip codes to trauma centers. Let set $\:i\in\:I$ represent the set of all zip codes located in the region of study which are the demand nodes for trauma services. These zip codes are located within a service region defined as a Trauma Service Area (TSA). Each TSA contains a set of designated TCCs. TCCs are classified based on the level of service they provide. Set $\:\mathcal{l}\in\:L$ is used to differentiate between two types of facilities given their level of service. $\:\mathcal{l}$=1 if the facility handles severe injuries (i.e., TCCs Level-I and Level-II) and $\:\mathcal{l}$=2 if the facility primarily manages non-severe injuries (i.e., TCCs Level-III and below) and function as intermediate facilities for stabilizing and transferring patients with more critical conditions. Every zip code in set $\:I$ has a daily demand per TCC type $\:\mathcal{l}$ represented by $\:{a}_{i\mathcal{l}}$.

The set of TCCs are represented by set $\:j\in\:J$. Some of the TCCs have aeromedical depots as part of their infrastructure and are termed as aeromedical depots. The aeromedical depots are represented by set $\:k\in\:K$. Clinical intervention is expected within a time $\:S$ from the moment of an injury incident. Demand region $\:i\:$is geographically covered by an ambulance if there exists a TCC $\:j$ with $\:{t}_{ij}^{G}\le\:S$, where $\:{t}_{ij}^{G}$ is the driving time from node $\:i$ to node $\:j$. Demand region $\:i\:$is geographically covered by an helicopter if there exists a TCC $\:j$ with $\:{t}_{ki}^{A}+{t}_{ij}^{A}{+t}_{\:}^{load}\le\:S$, where $\:{t}_{ki}^{A}$ denotes time taken to reach the demand node from the aeromedical depot $\:k$, $\:{t}_{ij}^{A}$ denotes the time taken to return to TCC $\:j$ from the demand node $\:i$,and $\:{t}_{\:}^{load}$ denotes the time required to load a patient into a helicopter. In this work the loading of the patient was set to 5 min as reported in [29]. The travel times were computed using ArcGIS Pro [33]. As a rule of thumb, $\:S$ is defined as 60 min. Due to the varying demands, the authors modeled the problem as stochastic which considers the uncertainty in the demand for trauma services per regional location. The objective is to find the locations of additional TCCs $\:j$ and aeromedical depots $\:k$ that will maximize the expected coverage demand within a time standard $\:S$ considering the randomness in the demand. Community and/or regional hospitals are not included when calculating coverage. However, these facilities are considered as part of the eligible TCC location set $\:J$.

## Formulation

In this section, a two-stage stochastic programming model, named the Stochastic Trauma System Configuration Problem (STSCP), is presented to model the problem as described in Sect. 3. The authors also present multiple modifications of the STSCP to address different scenarios for the problem. The decision variables, parameters, and sets used in the Stochastic Trauma System Configuration Problem (STSCP) are detailed in Table 1.

In the STSCP, “here and now” decisions (i.e., $\:{x}_{j\mathcal{l}}^{TC}$, $\:{x}_{k\mathcal{l}}^{AD},{z}_{kj\mathcal{l}}$) are made at the first stage before the realization of uncertain data. The uncertain data is represented by $\:\omega\:$, which is a scenario realization of the demand for trauma services in a specific location. In the second stage, after a realization of $\:\omega\:$ becomes known, a system configuration is generated by solving the appropriate optimization problem.

**Table 1:** Decision variables and parameters for proposed optimization model

Sets	
$\:I$	Set of injury demand nodes where $\:i\in\:I$ (patients in a geographical zone)
$\:J$	Set of eligible TCC locations where $\:j\in\:J$
$\:L$	Set of TCC types where $\:\mathcal{l}\in\:L\:$(classified based on their ability to treat severe injuries)
$\:K$	Set of eligible aeromedical depots (AD) locations where $\:k\in\:K$
$\:{N}_{i}$	$\:\left\{j|{t}_{ij}^{G}\le\:S\right\}=$ TCC sites within the time standard, $\:S$, of node $\:i$ by ground
$\:{M}_{i}$	$\:\left\{\left(j,k\right)|{t}_{ki}^{A}+{t}_{ij}^{A}{+t}_{\:}^{load}\le\:S\right\}=$ (AD, TCC) pairs within the time standard, $\:S$, of node $\:i$ by air
$\:{Q}_{j}$	$\:\left\{i|{t}_{ij}^{G}\le\:S\right\}=$ nodes $\:i$ within the time standard, $\:S$, of TCC site $\:j$
$\:{O}_{k}$	$\:\left\{i|{t}_{ki}^{A}+{t}_{ij}^{A}{+t}_{\:}^{load}\le\:S\right\}=$ nodes $\:i$ within the time standard, $\:S$, of AD site $\:k$
First Stage Decision Variables	
$\:{x}_{j\mathcal{l}}^{TC}$	= 1 if a TCC type $\:\mathcal{l}$ is sited at node $\:j$, 0 otherwise
$\:{x}_{k\mathcal{l}}^{AD}$	= 1 if an AD is sited at node $\:k$ with a type $\:\mathcal{l}$ trauma facility, 0 otherwise
$\:{z}_{kj\mathcal{l}}$	= 1 if an AD is sited at node $\:k$ and a TCC type $\:\mathcal{l}$ is sited at node $\:j$, 0 otherwise
Second Stage Decision Variables	
$\:{y}_{i\mathcal{l}}^{\omega\:}$	= 1 if demand for type $\:\mathcal{l}$ facility at node $\:i\:$under scenario $\:\omega\:$ is covered, 0 otherwise
$\:{v}_{i\mathcal{l}}^{\omega\:}$	= 1 if demand for type $\:\mathcal{l}$ facility at node $\:i$ under scenario $\:\omega\:$ is covered by ground, 0 otherwise
$\:{u}_{i\mathcal{l}}^{\omega\:}$	= 1 if demand for type $\:\mathcal{l}$ facility at node $\:i$ under scenario $\:\omega\:$ is covered by air, 0 otherwise
Parameters	
$\:S$	Time standard in minutes to provide care to patients suffering from a traumatic injury
$\:{p}_{\:}^{TC}$	The number of TCCs to be sited
$\:{p}_{\:}^{AD}$	The number of ADs to be sited
$\:{t}_{ij}^{G}$	The driving time from node $\:i$ to node $\:j$
$\:{t}_{ij}^{A}$	The flying time from node $\:i$ to node $\:j$
$\:{t}_{ki}^{A}$	The flying time from node $\:k$ to node $\:i$
$\:{t}_{\:}^{load}$	The loading time of patient into a helicopter
$\:{r}_{\mathcal{l}}$	Maximum number of trauma centers that can be place per type $\:l$
$\:{V}_{\mathcal{l}}^{min}$	Minimum volume of patients required to site a TCC type $\:\mathcal{l}$ at node $\:j$
$\:{V}_{\mathcal{l}}^{max}$	Maximum volume of patients allowed at TCC type $\:\mathcal{l}$ if sited at node $\:j$
$\:{V}_{AD}^{max}$	Maximum volume of severely injured patients ($\:\mathcal{l}=1)$ managed by AD if sited at node $\:k$
Stochastic Parameters	
$\:{a}_{i\mathcal{l}}^{\omega\:}$	Population demand for a TCC type $\:\mathcal{l}$ at node $\:i$ under scenario $\:\omega\:$

Equations $\:\left(1\right)$ to ($\:15)\:$describe the STSCP model. The objective function $\:\left(1\right)$ maximizes the expected patient coverage in a geographic area. The parameter $\:{p}_{\omega\:}$ represents the probability associated with scenario $\:\omega\:$, ensuring that the objective function accounts for the expected value across all possible scenarios in the model. The first stage of the model decides the system configuration in terms of where to locate TCCs and ADs. Constraint $\:\left(2\right)\:$limits the total number of trauma centers placed according to the value ($\:{p}_{\:}^{TC}$) which is provided by the decision maker. Constraint $\:\left(3\right)$ establishes that no more than one TCC can be placed in a node. The same limitations apply to ADs, as the total number of aeromedical depots placed cannot exceed the value ($\:{p}_{\:}^{AD}$), as established in constraint (4). Constraint (5) establishes that no more than one AD can be placed in a node. Constraints $\:\left(6\right)$ and $\:\left(7\right)$ check if a trauma facility or an aeromedical depot are located to each other or vice versa. The second stage of the model depicts a “scenario” for the problem. A scenario is defined as a representative demand for trauma services in the specified region. Hence, the evaluation of the trauma system configuration will be an aggregation of multiple scenarios. These scenarios are generated using forecasting methods from historical data [34]. Constraints $\:\left(8\right)$ - $\:\left(14\right)$ define the second stage of the model. Constraint $\:\left(8\right)$ determines if a demand node $\:i$ is covered by air or ground for scenario $\:\omega\:\in\:{\Omega\:}$. Constraint $\:\left(9\right)$ checks if there is a TCC that can cover demand at node $\:i$ by ground under scenario $\:\omega\:\in\:{\Omega\:}$ and constraint $\:\left(10\right)$ checks if there is an aeromedical depot that can cover demand at node $\:i$ by air under scenario $\:\omega\:\in\:{\Omega\:}$. Constraint $\:\left(11\right)$ places a limit as to the number of TCCs per type $\:\mathcal{l}$. Constraints $\:\left(12\right)$ and $\:\left(13\right)$ determine the volume of cases at each candidate location $\:j$ designated as a TCC, ensuring that the volume remains within the allowable bounds. The minimum bound reflects the recommendations from the American College of Surgeons, which aim to ensure the financial viability of a TCC. Each TCC must be able to offset the high costs associated with trauma readiness, including expenses for physicians, staff, equipment, and infrastructure. Constraint $\:\left(14\right)\:$determines the maximum volume of severely injured cases at each candidate AD location $\:k$, ensuring that the volume remains within the allowable bounds.

1 $$\:\text{max } z:\:\sum\:_{\omega\:\in\:{\Omega\:}}{p}_{\omega\:}*\left\{\sum\:_{i\in\:I}\sum\:_{\mathcal{l}\in\:L}{a}_{i\mathcal{l}}^{\omega\:}{y}_{i\mathcal{l}}^{\omega\:}\right\}$$

Subject to:

2 $$\:\sum\:_{j\in\:J}\sum\:_{\mathcal{l}\in\:L}{x}_{j\mathcal{l}}^{TC}\le\:\:{p}_{\:}^{TC}$$

3 $$\:\sum\:_{\mathcal{l}\in\:L}{x}_{j\mathcal{l}}^{TC}\le\:\:1,\:\:\:\forall\:j\in\:J$$

4 $$\:\sum\:_{k\in\:K}{\sum\:}_{\mathcal{l}\in\:L}{x}_{k\mathcal{l}}^{AD}\le\:{p}_{\:}^{AD}$$

5 $$\:\sum\:_{\mathcal{l}\in\:L}{x}_{k\mathcal{l}}^{AD}\le\:\:1,\:\:\:\forall\:k\in\:K$$

6 $$\:{z}_{kj\mathcal{l}}-{x}_{j\mathcal{l}}^{TC}\le\:0,\:\:\:\:\forall\:\:j\in\:J,\:\:\:\forall\:k\in\:K,\mathcal{\:}\forall\:\mathcal{l}\in\:L$$

7 $$\:{z}_{kj\mathcal{l}}-{x}_{k\mathcal{l}}^{AD}\le\:0,\:\:\:\:\forall\:\:j\in\:J,\:\:\:\forall\:k\in\:K,\mathcal{\:}\forall\:\mathcal{l}\in\:L$$

8 $$\:{y}_{i\mathcal{l}}^{\omega\:}-{v}_{i\mathcal{l}}^{\omega\:}-{u}_{i\mathcal{l}}^{\omega\:}\le\:0,\:\:\:\forall\:i\in\:I,\mathcal{\:}\forall\:\mathcal{l}\in\:L,\:\:\forall\:\omega\:\in\:{\Omega\:}$$

9 $$\:{v}_{i\mathcal{l}}^{\omega\:}-{\sum\:}_{j\in\:{N}_{i}}^{}{x}_{j\mathcal{l}}^{TC}\le\:0,\:\:\:\forall\:i\in\:I,\mathcal{\:}\forall\:\mathcal{l}\in\:L,\:\:\forall\:\omega\:\in\:{\Omega}$$

10 $$\begin{array}{lc}u_{i\mathcal l}^{\omega\:}-{\textstyle\sum_{\left(j,k\right)\in\:M_i}}z_{kj\mathcal l}\leq\:0,\:\:\:\forall\:i\in\:I,\:\mathcal l=1\:,\\\forall\:j\in\:J,\:\:k\in\:K,\:\:\forall\:\omega\:\in\:\Omega\end{array}$$

11 $$\:{\sum\:}_{j\in\:J}{x}_{j\mathcal{l}}^{TC}\:\:\le\:\:{r}_{\mathcal{l}},\:\:\:\forall\:\mathcal{l}\in\:L$$

12 $$\:\sum\:_{i\in\:{Q}_{j}}{a}_{i\mathcal{l}}^{\omega\:}\:{x}_{j\mathcal{l}}^{TC}\ge\:{V}_{\mathcal{l}}^{min}, \;\;\;\forall\:\:j\in\:J,\mathcal{\:}\mathcal{\:}\forall\:\mathcal{l}\in\:L,\:\forall\:\omega\:\in\:{\Omega\:}$$

13 $$\:\sum\:_{i\in\:{Q}_{j}}{a}_{i\mathcal{l}}^{\omega\:}\:{x}_{j\mathcal{l}}^{TC}\le\:{V}_{\mathcal{l}}^{max}, \;\;\;\forall\:\:j\in\:J,\mathcal{\:}\mathcal{\:}\forall\:\mathcal{l}\in\:L,\:\forall\:\omega\:\in\:{\Omega\:}$$

14 $$\:{\sum\:}_{i\in\:{O}_{k}}^{\:}{{a}_{i\mathcal{l}}^{\omega\:}\:x}_{k\mathcal{l}}^{AD}\le\:{V}_{AD}^{max}, \:\:\:\forall\:k\in\:K,\:\:\mathcal{l}=1,\:\forall\:\omega\:\in\:{\Omega\:}$$

15 $$\:{x}_{j\mathcal{l}}^{TC},\:{x}_{k\mathcal{l}}^{AD},\:{z}_{kj\mathcal{l}},\:{y}_{i\mathcal{l}}^{\omega\:},\:{v}_{i\mathcal{l}}^{\omega\:},\:{u}_{i\mathcal{l}}^{\omega\:}=\left\{\text{0,1}\right\}$$

The candidate sites for locating new TCCs are known and finite. In this work, TCC candidate locations are those associated with existing hospitals in the TSA including those not classified as TCCs. The availability of ground ambulances for the transportation of patients from location $\:i$ to a TCC $\:j$ is assumed to be unlimited. Finally, TCC coverage is defined as all zip-codes $\:i$ within $\:S$ as explained in Sect. 3. To solve the two-stage stochastic programming model, it is reformulated as its deterministic equivalent [35]. The model is then implemented in AMPL and solved using CPLEX [36], which efficiently handles large-scale mixed-integer and linear optimization problems.

## Application

The state of Texas plans to expand the availability of TCCs in certain state areas [6]. The state identified the limited availability of high-level trauma care (i.e., type $\:\mathcal{l}=1$) in rural areas when compared to trauma facilities in densely populated urban areas. The stochastic programming model formulated in the previous section was implemented and applied to one of the trauma service areas of the state of Texas. A series of experiments are presented in the following sections with the goal of assessing the level of coverage obtained by the solutions provided by the model under different conditions. Two strategies (i.e., construction and improvement), for the trauma network design, are considered in this study. The *construction strategy* considers an empty system, and the goal is to build the entire trauma network. The *improvement strategy* considers the existing trauma network, and the goal is to add additional capacity to the trauma network. Separate models were formulated to address both strategies, and their performance is compared against the benchmark network.

The stochastic programming models were implemented in AMPL and solved using ILOG CPLEX. The traveling times needed to define some of the parameters were computed using ArcGIS Pro [33] which has a road network database, and the values are a result of calculations based on real time speed limits (helicopter speed was assumed to be 120 mph). The distances from zip codes $\:i\in\:I$ to TCC candidate locations $\:j\in\:J$ were computed using geocoded centroids that represent the population for the zip code.

### Real trauma service area

**Fig. 1:**
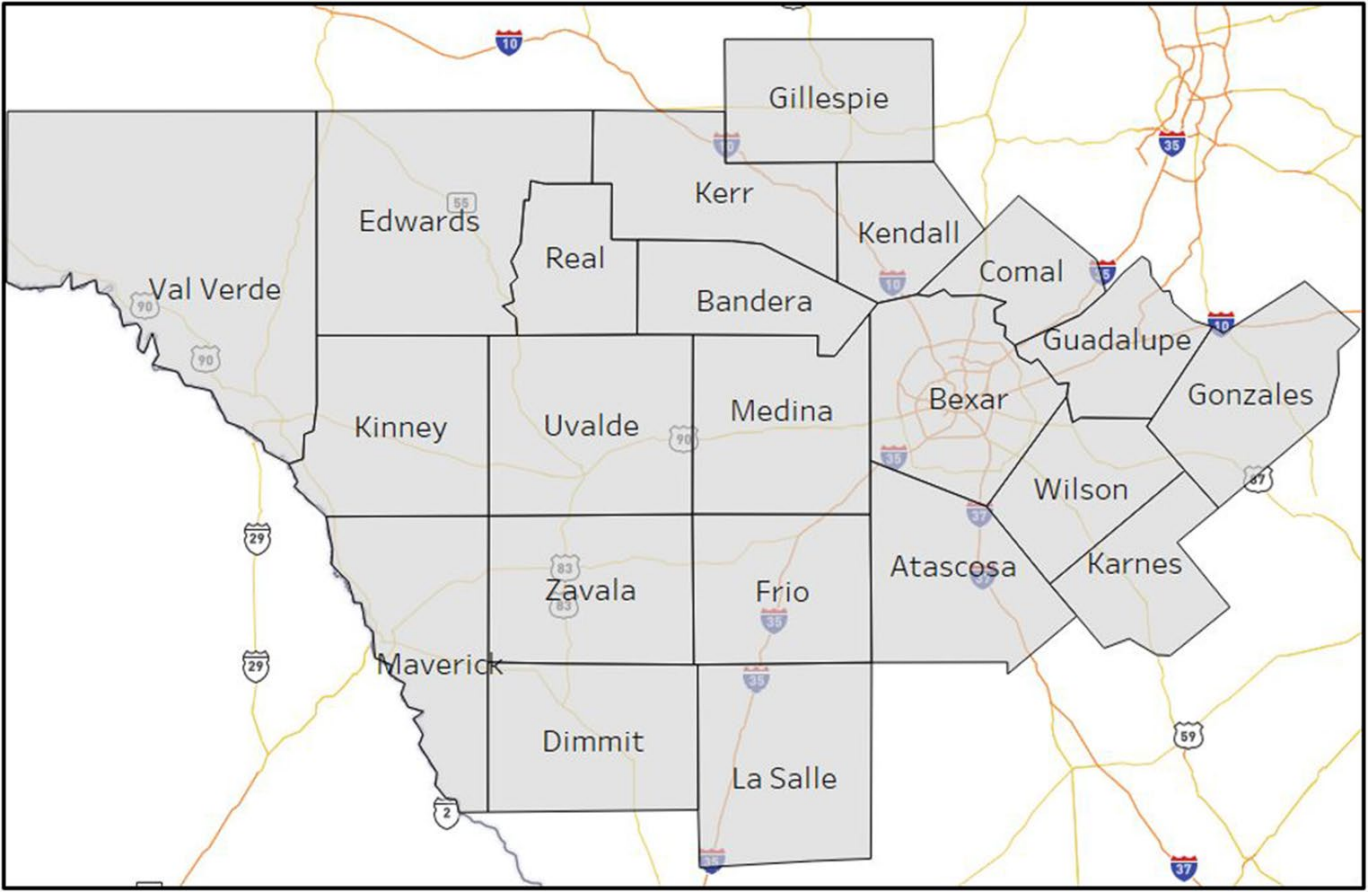
Counties located in Trauma Service Area (TSA) $\:P$

**Fig. 2:**
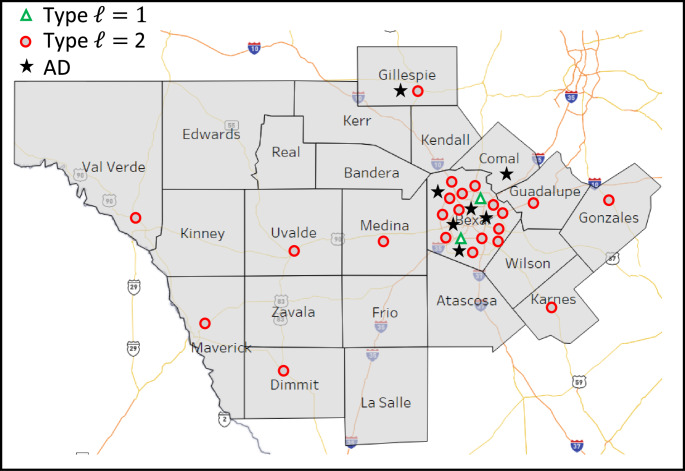
TCCs and ADs in trauma Service Area (TSA) *P*

The trauma network in the state of Texas serves over twenty-eight million citizens. The Texas DSHS had divided the state into 22 trauma service areas (TSA) [37]. The authors chose TSA$\:P$ for this study as it encompasses a mix of densely populated urban areas in addition to rural areas with lower population densities. 17 of the 22 counties in TSA *P* are classified as rural. This region provides a test base for the models to determine trauma coverage under varied service demands. Figure 1 depicts the counties within TSA $\:P$. Figure 2 shows the locations of the twenty-four hospitals designated as TCCs and seven aeromedical depots. The triangles depict type $\:\mathcal{l}=1$ (i.e., Level-I & Level-II TCCs), and the circles depict type $\:\mathcal{l}=2$ (i.e., Level-III and below TCCs) facilities. The star icon represents the location of aeromedical depots. The trauma network presented in Fig. 2 is the benchmark network considered in the computational study. The TSA area includes 176 unique zip codes, twenty-four designated TCCs and seven aeromedical depots.

The Texas DSHS provided two years of data, 2015 and 2016, for trauma records and emergency medical services (EMS) records. The datasets include detailed information on trauma incidents in Texas and the corresponding EMS. In 2015, the trauma records documented a total of 82,292 trauma incidents, while the EMS records reported a total of 1,744,385 incidents. In 2016, the trauma records documented a total of 75,796 incidents, while the Emergency Medical Databases documented 1,571,732 incidents. The 2020 data for trauma incidents not related to COVID-19 was unavailable. However, during the COVID-19 pandemic, the Texas Department of State Health Services (DSHS) maintained a comprehensive dashboard that reported COVID-19 cases daily. The data used to create this dashboard was shared with the authors and was utilized to forecast the demand for COVID-19 trauma-linked cases.

### Experimental setup

The stochastic programming models were formulated based on the data provided by the Texas DSHS. The models consider a network of hospitals with twenty-four designated TCCs, seven aeromedical depots, and sixty-two hospitals that are not designated as TCCs. ArcGIS Pro was used to obtain the actual ground times and airtimes from each incident location to all hospital sites. Incident locations were represented using zip codes and a total of 176 zip codes are considered in this study. The resulting time matrices, one each for ground and air (176 × 86 cells each), served to define sets $\:{N}_{i}$ and $\:{M}_{i}$.

The scenarios for the stochastic programming models are generated using forecasting models that are based on the historical data for every zip code. To forecast trauma injury volumes, five time-series forecasting models were evaluated: Moving Average, Exponentially Weighted Moving Average (EWMA), Holt’s Method (Additive Trend), Winter’s Method (Additive Trend and Seasonality), and Autoregressive Integrated Moving Average (ARIMA). The computational results are reported in Joshi et al. [34]. These models were applied to historical trauma injury data obtained from the Texas DSHS, with the selection of the best-performing model for each region based on forecast accuracy, measured using the Mean Absolute Percentage Error (MAPE). ARIMA and EWMA consistently provided the most accurate forecasts, with ARIMA excelling in capturing long-term temporal patterns and EWMA proving effective for short-term predictions in regions with high variability. The scenarios for the stochastic programming models are generated using these forecasting models, which rely on historical data for each zip code. Each scenario comprises the daily demand for trauma services at the zip code level. By leveraging these models, we aim to provide reliable forecasts that can inform resource allocation and infrastructure planning for trauma care services in rural areas. A scenario comprises the daily demand for trauma services per trauma for every zip code. Scenarios 1 to 3 can be termed as “*normal demand”* and scenarios 4 as “*COVID-19 demand”*. The computational study considers four problem instances.

- **Instance 1 (IN1) - Normal operating conditions**: the probability for COVID-19 scenario is zero (i.e., $\:{p}_{\omega\:}=0$ for $\:\omega\:=4$).
- **Instance 2 (IN2) - COVID-19 pandemic conditions**: the probability for COVID-19 scenario is 50%.
- **Instance 3 (IN3) - COVID-19 pandemic conditions**: the probability for COVID-19 scenario is 25%.
- **Instance 4 (IN4) - COVID-19 pandemic conditions**: the probability for COVID-19 scenario is 75%.

Table 2 lists the scenario probabilities for all problem instances (i.e., IN1, IN2, IN3, and IN4).

**Table 2:** Scenario probabilities for stochastic programming model

Instance #	Probability per scenario $\:\varvec{\omega\:}$			
	$\:\varvec{\omega\:}=1$ 2015 demand	$\:\varvec{\omega\:}=2$ 2016 demand	$\:\varvec{\omega\:}=3$ 2-year average demand	$\:\varvec{\omega\:}=4$ 2020 - COVID-19 demand
IN1	0.33	0.33	0.33	0.00
IN2	0.16	0.16	0.16	0.50
IN3	0.25	0.25	0.25	0.25
IN4	0.083	0.083	0.083	0.75

The candidate sites for locating TCCs are known and finite. We considered the existing locations of hospitals (both TCCs and non-TCCs) serving TSA *P* as candidate sites for TCC and AD placement. Out of the 24 TCCs, 2 were classified as $\:\mathcal{l}$=1 which treat severe injuries (ISS > 15) and 22 were classified as $\:\mathcal{l}$=2 which treat non-severe injuries (ISS ≤ 15).

The computational study examines three variants of the STSCP model, defined by equations (1) to (15): (1) Benchmark Model (BM), (2) Construction Model (CM), and (3) Improvement Model (IM). The BM assesses the performance of the existing trauma network. The CM starts with an empty system, where the locations of existing TCCs are not fixed and there are no limitations on the number of TCCs per level. The IM assumes an existing trauma network and aims to expand it by determining where to locate additional TCCs to improve patient access. Table 3 provides a mathematical explanation of the three model variants studied in this paper. Parameters $\:{p}_{\:}^{TC}$and $\:{p}_{\:}^{AD}$were assigned based on the TSA *P* network, 24 and 7 respectively. The values for parameters $\:{V}_{\mathcal{l}}^{min}$, $\:{V}_{\mathcal{l}}^{max}$, and $\:{V}_{AD}^{max}$ were set as 1, 5, and 7 per day. The value assign to $\:{V}_{\mathcal{l}}^{min}$ aims to meet the minimum bound recommended by the American College of Surgeons of at least 200 per year. The values assigned to $\:{V}_{\mathcal{l}}^{max}$, and $\:{V}_{AD}^{max}$ were based on the data obtained from the Texas DSHS. For the CM, we experimented with the number of TCCs and AD and incremented them gradually until we found the combination that provided the maximum coverage with the minimum number of facilities. In the IM, we explored various combinations by incrementally adding TCCs of different types and aeromedical depots to enhance trauma coverage. We reported the combinations that provided the best results. Table 3 discusses the additional parameters and constraint modifications needed to implement IM.

**Table 3:** TCC system set-up

Model name	STSCP Modification	Description
Benchmark Model (BM)	• Set $\:{x}_{j1}^{TC}=1,\:\:\forall\:j\in\:{\widehat{J}}_{1}$ and $\:{x}_{j2}^{TC}=1,\:\:\:\forall\:j\in\:{\widehat{J}}_{2}$ to fix the location of existing TCCs. In this model, set $\:{\widehat{J}}_{1}$ represents the TCCs type $\:\mathcal{l}$=1 that are currently located in the TCC network, $\:{\widehat{J}}_{1}\subset\:J$. Also, set $\:{\widehat{J}}_{2}$ represents the TCCs type $\:\mathcal{l}$=2 that are currently located in the trauma network, $\:{\widehat{J}}_{2}\subset\:J$. • Set $\:\widehat{K}$ represents the aeromedical depots that are currently located in the trauma network, $\:\widehat{K}\subset\:K$.	Assess the performance of an existing trauma network
Construction Model (CM)	• The CM model removes constraint $\:\left(1k\right)$ which limits the number of TCCs that can be placed per type $\:\mathcal{l}$.	Consider an empty system where none of the TCCs are fixed to a location.
Improvement Model (IM)	• Set $\:{x}_{j1}^{TC}=1,\:\:\forall\:j\in\:{\widehat{J}}_{1}$ and $\:{x}_{j2}^{TC}=1,\:\:\:\forall\:j\in\:{\widehat{J}}_{2}$ to fix the location of existing TCCs. In this model, set $\:{\widehat{J}}_{1}$ represents the TCCs type $\:\mathcal{l}$=1 that are currently located in the TCC network, $\:{\widehat{J}}_{1}\subset\:J$. Also, set $\:{\widehat{J}}_{2}$ represents the TCCs type $\:\mathcal{l}$=2 that are currently located in the trauma network, $\:{\widehat{J}}_{2}\subset\:J$. • Set $\:\widehat{K}$ represents the aeromedical depots that are currently located in the trauma network, $\:\widehat{K}\subset\:K$. • Three new parameters are defined $\:{\alpha\:}^{TC}$, $\:{\alpha\:}^{AD}$, and $\:{\mu\:}_{\mathcal{l}}^{\:}$ o $\:{\alpha\:}^{TC}$= number of additional TCCs to be added to the trauma network o $\:{\alpha\:}^{TC}$= number of additional aeromedical depots to be added to the trauma network o $\:{\mu\:}_{\mathcal{l}}^{\:}$ = number of additional TCCs to be added to the trauma network per type $\:\mathcal{l}$ • Constraint (2) is updated to $\:\sum\:_{j\in\:J}\sum\:_{\mathcal{l}\in\:L}{x}_{j\mathcal{l}}^{TC}\le\:\:{p}_{\:}^{TC}+\:{\alpha\:}^{TC}$ • Constraint (4) is updated to $\:\sum\:_{k\in\:K}{\sum\:}_{\mathcal{l}\in\:L}{x}_{k\mathcal{l}}^{AD}\le\:{p}_{\:}^{AD}+\:{\alpha\:}^{AD}$ • Constraint (11) is updated to $\:{\sum\:}_{j\in\:J}{x}_{j\mathcal{l}}^{TC}\:\le\:\:{r}_{\mathcal{l}}+{\mu\:}_{\mathcal{l}}^{\:},\:\:\:\forall\:\mathcal{l}\in\:L$	Similar to BM since its starts with an existing trauma network. However, this model considers the expansion of the trauma network by deciding where to locate additional TCCs and aeromedical depots.

## Computational study

We now report computational results to evaluate the performance of the IM and CM models. The results were compared with the BM, for each of the four problem instances listed in Table 2. Tables 4, 5 and 6 present the results for the experimental setup presented in the previous section. Table 4 presents the percentage coverage per county under BM, CM, and IM. Table 4 lists the TCC hospitals (i.e., type $\:\mathcal{l}$= 1and type $\:\mathcal{l}$= 2) and aeromedical depots located per county under the CM and compares the TCC hospitals located against the BM. Table 4 identifies counties that are classified as rural in **bold**. Table 5 lists the TCC hospitals (i.e., type $\:\mathcal{l}$= 1and type $\:\mathcal{l}$= 2) and aeromedical depots located per county under the IM and compares the TCC hospitals located against the BM.

Table 4 indicates that the CM model offers comprehensive coverage for most counties under normal and COVID-19 operating conditions. However, the CM model solution failed to achieve full coverage (100%) for four counties under COVID-19 operating conditions: Edwards, La Salle, Real, and Val Verde. This shortfall occurred because fixed locations were used for TCCs and ADs, resulting in some demand points falling outside the 60-minute coverage area (i.e. parameter $\:S$). The CM was able to provide solutions that improved access to trauma care in eight rural counties including Dimmit, Frio, Gillespie, Gonzales, Karnes, Kinney, Uvalde, and Zavala.

**Table 4:** Percentage coverage per County under BM, CM, and IM

County	IN1-Normal			COVID-19 IN2-50%			COVID-19 IN3-25%			COVID-19 IN4-75%		
	BM	CM	IM	BM	CM	IM	BM	CM	IM	BM	CM	IM
Atascosa	100	100	100	100	100	100	100	100	100	100	100	100
Bandera	100	100	100	100	100	100	100	100	100	100	100	100
Bexar	100	100	100	100	100	100	100	100	100	100	100	100
Comal	100	100	100	100	100	100	100	100	100	100	100	100
Dimmit	100	100	100	88	100	100	88	100	100	88	100	100
Edwards	100	100	100	88	88	88	88	88	88	88	88	88
Frio	100	100	100	84	100	100	84	100	100	84	100	100
Gillespie	67	100	100	63	100	100	63	100	100	63	100	100
Gonzales	100	100	100	88	100	100	88	100	100	88	100	100
Guadalupe	100	100	100	100	100	100	100	100	100	100	100	100
Karnes	100	100	100	88	100	100	88	100	100	88	100	100
Kendall	100	100	100	100	100	100	100	100	100	100	100	100
Kerr	67	100	100	64	100	100	64	100	100	64	100	100
Kinney	100	100	100	88	100	88	88	100	88	88	100	88
La Salle	67	100	100	50	96	96	50	96	96	50	96	96
Maverick	67	100	67	63	100	63	63	100	63	63	100	63
Medina	100	100	100	100	100	100	100	100	100	100	100	100
Real	100	100	100	88	94	88	88	94	88	88	94	94
Uvalde	100	100	100	88	100	100	88	100	100	88	100	100
Val Verde	67	67	67	63	63	63	63	63	63	63	63	63
Wilson	100	100	100	100	100	100	100	100	100	100	100	100
Zavala	67	100	100	63	100	100	63	100	100	63	100	100

**Fig. 3:**
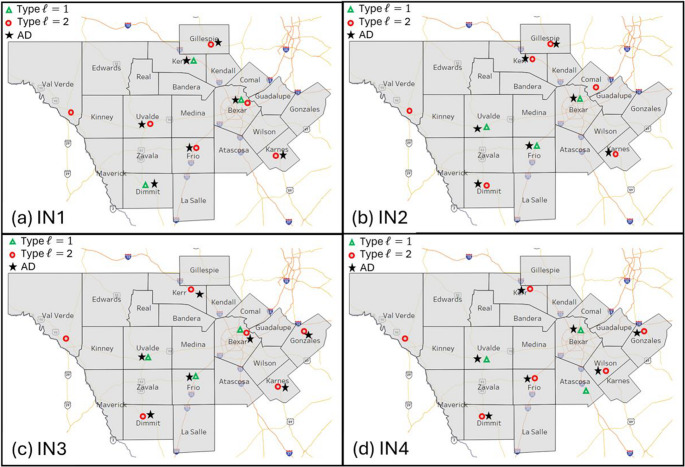
Construction Model (CM) TCC hospitals and ADs placement per instance (IN), (**a**) IN1: probability of COVID-19 demand = 0%,(**b**) IN2: probability of COVID-19 demand = 50%, (**c**) IN3: probability of COVID-19 demand = 25%, and (**d**) IN1: probability of COVID-19 demand = 75%

Table 5 compares the location of the TCCs for CM against BM. Remember that CM considers an empty system where none of the TCCs are fixed to a location. Therefore, the results show a higher coverage for CM with fewer TCC facilities. For instance, only six type $\:\mathcal{l}$=2 TCCs are needed under CM versus twenty-two under BM. The other major difference between CM and BM is the location of the type $\:\mathcal{l}$=1 TCC facilities as illustrated in Fig. 3. Instead of placing all the TCC type $\:\mathcal{l}$=1 facilities in the Bexar county (i.e., San Antonio – high population density) as illustrated in Fig. 2, the CM model placed the TCCs in several counties within the studied region. For instance, under normal operations without COVID-19 demand (i.e., Fig. 3a) one TCC type $\:\mathcal{l}$=1 was located in the north side of the region, Kerr county, and one was located in the south side of the region, Dimmit county. On the other hand, under COVID-19 demand (i.e., Fig. 3b and c, and 3c), the TCCs type $\:\mathcal{l}$=1 were located counties closer to Bexar county where a higher population density exists.

**Table 5:** Construction model (CM) TCC hospitals and aeromedical depots (AD) located versus benchmark model (BM)

County	BM Benchmark			IN1: CM COVID19-0%			IN2: CM COVID19-50%			IN3: CM COVID19-25%			IN4: CM COVID19-75%		
	type $\:\mathcal{l}$= 1	type $\:\mathcal{l}$= 2	AD	type $\:\mathcal{l}$= 1	type $\:\mathcal{l}$= 2	AD	type $\:\mathcal{l}$= 1	type $\:\mathcal{l}$= 2	AD	type $\:\mathcal{l}$= 1	type $\:\mathcal{l}$= 2	AD	type $\:\mathcal{l}$= 1	type $\:\mathcal{l}$= 2	AD
Atascosa	0	0	0	0	0	0	0	0	0	0	0	0	**1**	0	0
Bandera	0	0	0	0	0	0	0	0	0	0	0	0	0	0	0
Bexar	**2**	**13**	**5**	**1**	**1**	**1**	**1**	0	**1**	**1**	**1**	**1**	**1**	0	**1**
Comal	0	0	**1**	0	0	0	0	**1**	0	0	0	0	0	0	0
Dimmit	0	**1**	0	**1**	0	**1**	0	**1**	**1**	0	**1**	**1**	0	**1**	**1**
Edwards	0	0	0	0	0	0	0	0	0	0	0	0	0	0	0
Frio	0	0	0	0	**1**	**1**	**1**	0	**1**	**1**	0	**1**	0	**1**	**1**
Gillespie	0	**1**	**1**	0	**1**	**1**	0	**1**	**1**	0	0	0	0	0	0
Gonzales	0	**1**	0	0	0	0	0	0	0	0	**1**	**1**	0	**1**	**1**
Guadalupe	0	**1**	0	0	0	0	0	0	0	0	0	0	0	0	0
Karnes	0	**1**	0	0	**1**	**1**	0	**1**	**1**	0	**1**	**1**	0	0	0
Kendall	0	0	0	0	0	0	0	0	0	0	0	0	0	0	0
Kerr	0	0	0	**1**	0	**1**	0	**1**	**1**	0	**1**	**1**	0	**1**	**1**
Kinney	0	0	0	0	0	0	0	0	0	0	0	0	0	0	0
La Salle	0	0	0	0	0	0	0	0	0	0	0	0	0	0	0
Maverick	0	**1**	0	0	0	0	0	0	0	0	0	0	0	0	0
Medina	0	**1**	0	0	0	0	0	0	0	0	0	0	0	0	0
Real	0	0	0	0	0	0	0	0	0	0	0	0	0	0	0
Uvalde	0	**1**	0	0	**1**	**1**	**1**	0	**1**	**1**	0	**1**	**1**	0	**1**
Val Verde	0	**1**	0	0	**1**	0	0	**1**	0	0	**1**	0	0	**1**	0
Wilson	0	0	0	0	0	0	0	0	0	0	0	0	0	**1**	**1**
Zavala	0	0	0	0	0	0	0	0	0	0	0	0	0	0	0
Totals	**2**	**22**	**7**	**3**	**6**	**7**	**3**	**6**	**7**	**3**	**6**	**7**	**3**	**6**	**7**

Next we compare the IM against the BM model. Remember that IM fixes the location of the TCCs in the BM. However, the IM model considers the expansion of the trauma network by deciding where to locate additional type $\:\mathcal{l}$=1 and type $\:\mathcal{l}$=2 TCCs and aeromedical depots. In this computational study we allowed up to three additional type $\:\mathcal{l}$=1, type $\:\mathcal{l}$=2 TCCs, and Ads. As expected, the results show higher coverage for IM when compared to BM model. For instance, instead of just two type $\:\mathcal{l}$=1 TCCs facilities, the IM requires five for all instances as reported in Table 6. The other major difference is the additional aeromedical depots needed by IM, instead of the original seven, it requires ten.

Table 4 indicates that the IM model offers comprehensive coverage for most counties under normal and COVID-19 operating conditions. However, the IM model solution failed to achieve full coverage (100%) for six counties under COVID-19 operating conditions: Edwards, Kinney, La Salle, Maverick, Real, and Val Verde. This shortfall occurred because fixed locations were used for TCCs and ADs, resulting in some demand points falling outside the 60-minute coverage area (i.e. parameter $\:S$). The IM was able to provide solutions that improved access to trauma care in eight rural counties including Dimmit, Frio, Gillespie, Gonzales, Karnes, Kerr, Uvalde, and Zavala.

**Fig. 4:**
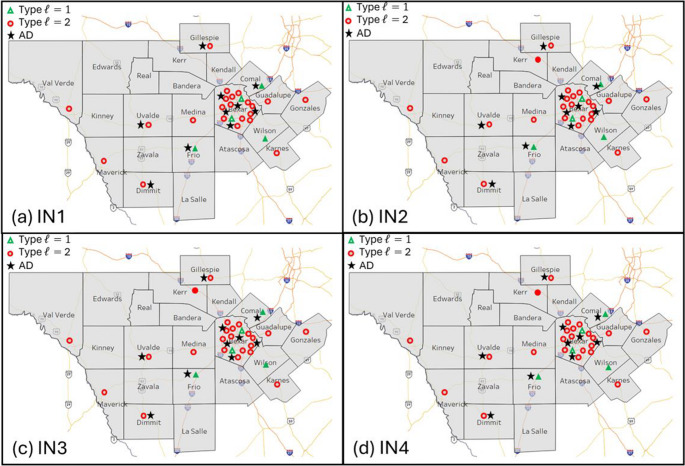
Improvement Model (IM) TCC hospitals and ADs placement per instance (IN), (**a**) IN1: probability of COVID-19 demand = 0%,(**b**) IN2: probability of COVID-19 demand = 50%, (**c**) IN3: probability of COVID-19 demand = 25%, and (d) IN1: probability of COVID-19 demand = 75%

Table 6 compares the location of the TCCs for IM against BM. The number of type $\:\mathcal{l}$=2 TCCs did not change much under IM versus BM. The major difference is the addition of one additional type $\:\mathcal{l}$=2 in Kerr county for instances IN2, IN3, and IN4 when COVID-19 demand is expected. The other major difference between IM and BM is the location of the type $\:\mathcal{l}$=1 TCC facilities as illustrated in Fig. 4. Instead of placing all the TCC type $\:\mathcal{l}$=1 facilities in the Bexar county (i.e., San Antonio – high population density) as illustrated in Fig. 2, the IM model placed the three additional TCCs in three counties within the studied region. The additional TCCs type $\:\mathcal{l}$=1 were located counties closer to Bexar county where a higher population density exists.

**Table 6:** Improvement model (IM) TCC hospitals and aeromedical depots located versus benchmark model (BM)

County	BM Benchmark			IN1: IM COVID19-0%			IN2: IM COVID19-50%			IN3: IM COVID19-25%			IN4: IM COVID19-75%		
	type $\:\mathcal{l}$= 1	type $\:\mathcal{l}$= 2	AD	type $\:\mathcal{l}$= 1	type $\:\mathcal{l}$= 2	AD	type $\:\mathcal{l}$= 1	type $\:\mathcal{l}$= 2	AD	type $\:\mathcal{l}$= 1	type $\:\mathcal{l}$= 2	AD	type $\:\mathcal{l}$= 1	type $\:\mathcal{l}$= 2	AD
Atascosa	0	0	0	0	0	0	0	0	0	0	0	0	0	0	0
Bandera	0	0	0	0	0	0	0	0	0	0	0	0	0	0	0
Bexar	**2**	**13**	**5**	**2**	**13**	**5**	**2**	**13**	**5**	**2**	**13**	**5**	**2**	**13**	**5**
Comal	0	0	**1**	**1**	0	**1**	**1**	0	**1**	**1**	0	**1**	**1**	0	**1**
Dimmit	0	**1**	0	0	**1**	**1**	0	**1**	**1**	0	**1**	**1**	0	**1**	**1**
Edwards	0	0	0	0	0	0	0	0	0	0	0	0	0	0	0
Frio	0	0	0	**1**	0	**1**	**1**	0	**1**	**1**	0	**1**	**1**	0	**1**
Gillespie	0	**1**	**1**	0	**1**	**1**	0	**1**	**1**	0	**1**	**1**	0	**1**	**1**
Gonzales	0	**1**	0	0	**1**	0	0	**1**	0	0	**1**	0	0	**1**	0
Guadalupe	0	**1**	0	0	**1**	0	0	**1**	0	0	**1**	0	0	**1**	0
Karnes	0	**1**	0	0	**1**	0	0	**1**	0	0	**1**	0	0	**1**	0
Kendall	0	0	0	0	0	0	0	0	0	0	0	0	0	0	0
Kerr	0	0	0	0	0	0	0	**1**	0	0	**1**	0	0	**1**	0
Kinney	0	0	0	0	0	0	0	0	0	0	0	0	0	0	0
La Salle	0	0	0	0	0	0	0	0	0	0	0	0	0	0	0
Maverick	0	**1**	0	0	**1**	0	0	**1**	0	0	**1**	0	0	**1**	0
Medina	0	**1**	0	0	**1**	0	0	**1**	0	0	**1**	0	0	**1**	0
Real	0	0	0	0	0	0	0	0	0	0	0	0	0	0	0
Uvalde	0	**1**	0	0	**1**	**1**	0	**1**	**1**	0	**1**	**1**	0	**1**	**1**
Val Verde	0	**1**	0	0	**1**	0	0	**1**	0	0	**1**	0	0	**1**	0
Wilson	0	0	0	**1**	0	0	**1**	0	0	**1**	0	0	**1**	0	0
Zavala	0	0	0	0	0	0	0	0	0	0	0	0	0	0	0
Totals	**2**	**22**	**7**	**5**	**22**	**10**	**5**	**23**	**10**	**5**	**23**	**10**	**5**	**23**	**10**

**Fig. 5:**
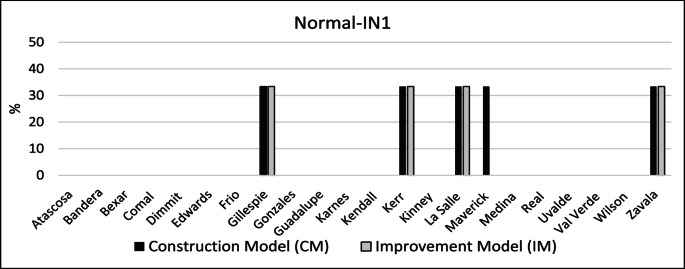
Coverage improvement for all counties – no COVID-19

Figure 5 depicts the percentage of coverage increase per county for CM and the IM when compared to the BM under normal conditions. The results indicate that, under normal operating conditions (i.e., IN1), both the CM and IM increased coverage by 33% in Gillespie, Kerr, La Salle, and Zavala counties. All of those counties, with the exception of Kerr County, are rural. In addition, CM increased coverage of Maverick County by 33%.

**Fig. 6:**
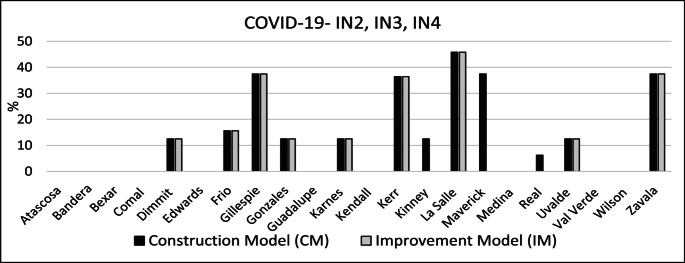
Coverage improvement for all counties – COVID-19

Figure 6 illustrates the percentage of coverage increase per county relative to the BM for CM and IM under COVID-19 conditions (i.e., IN2, IN3, and IN4). Under both the CM and IM, La Salle, a rural county demonstrates a significant improvement of 45%. CM and IM coverage increased by 37% in Gillespie, Kerr and Zavala counties. Those counties, except for Kerr County, are rural. The following rural counties obtained a coverage increase of over 10% under CM and IM: Dimmit, Frio, Gonzales, Karnes and Uvalde. Under CM the following counties obtained coverage increases Kinney and Real.

## Conclusions

The goal of this research is to derive new models for the design and/or expansion of a trauma care system within a specified area. The optimal geographic configuration of TCCs is key to maximizing accessibility while promoting the efficient use of resources. The aim of this paper is to report on the development of a two-stage stochastic optimization model for geospatial expansion of a trauma network in the state of Texas considering the COVID-19 pandemic.

A two-stage stochastic programming model was introduced along with its iterations of which the latter are modified versions of the original model that are used to represent modified networks of the current trauma network to determine their coverage. The current trauma network was termed as the benchmark model, the network to evaluate all proposed networks against. The construction model develops a new network with a wider assortment of hospitals and medical centers available at its disposal. The improvement model adds TCCs and aeromedical depots. The improvement model was developed to determine changes in coverage when facilities were added incrementally to the current trauma network and observe improvements. The models are useful to provide insight into key factors such as demand, patient coverage, and TCCs placement. Specifically, the authors were interested in studying the impact of the COVID-19 and/or similar pandemics in the future expansion of the trauma network.

When considering population coverage, the construction model provided the highest coverage percentage across all counties with the minimum number of facilities. However, this method is not practical unless you have the flexibility to build the network of TCCs from scratch. The improvement model also provides high overall population coverage. The incremental increase of facilities in the benchmark network produces significant improvement in coverage which is a more pragmatic approach to coverage improvement rather than the complete restructuring of the benchmark network.

The research presented in this paper offers substantial benefits for rural counties. Rural areas often face significant challenges in accessing timely and adequate trauma care due to factors such as distance, limited resources, and lower population densities. The models developed in this study specifically address these challenges by optimizing the placement of trauma care centers and aeromedical depots to ensure that even the most remote areas are covered within the critical time window for trauma care.

- Improved Accessibility: The optimized geographic configuration of trauma care centers ensures that rural populations have better access to high-quality trauma care. This is particularly important in emergencies where timely intervention can significantly impact patient outcomes.
- Efficient Resource Utilization: By strategically placing trauma care centers and aeromedical depots, the models promote the efficient use of resources, ensuring that rural areas are not left underserved. This helps in maximizing the reach of existing facilities and improving overall healthcare delivery.
- Enhanced Emergency Preparedness: The inclusion of aeromedical depots in the trauma network design enhances emergency preparedness in rural areas. Helicopter transport can bridge the gap created by long distances and difficult terrains, ensuring rapid response and patient transfer to appropriate facilities.
- Equitable Healthcare: The research emphasizes the importance of equitable access to trauma care, ensuring that rural populations receive the same level of care as urban populations. This helps in reducing disparities in healthcare access and outcomes between different geographic regions.
- Adaptability to Unexpected Events: The models consider the impact of unexpected events such as pandemics, which can strain healthcare systems. By incorporating flexibility and scalability, the trauma network can adapt to varying demands, ensuring continuous and reliable care for rural communities.

In conclusion, the work presented in this paper not only advances the field of trauma care optimization but also provides tangible benefits for rural counties, enhancing their access to critical healthcare services and improving overall public health outcomes.

## Recommendations and future research prospects

The next evolutionary stage of this model is to be scaled up to cover more land, in terms of counties. Entire states with their trauma networks can be mapped and analyzed to determine their performance and recommend strategies for either expansion or re-designation. A desired scenario would be to cover the state of Texas to determine its overall population coverage. This model can theoretically be scaled up to cover a country provided the necessary computing power and exact databases for distances are available.

There are several additions to the decision-making model that can improve the outcome significantly. The model does not include characteristics of the facilities themselves such as bedding capacity, available personnel, and equipment available. The model can be tailored to plan medical services in disaster prone areas to prepare for inevitable natural calamities so that administrations are prepared to meet the challenges of providing rapid and accessible care to residents. Additions that involve micro-management of facilities that are combined with macro-based factors will undoubtedly improve the decision-making process of the model proposed.

## Figures and Tables

**Table 7:**
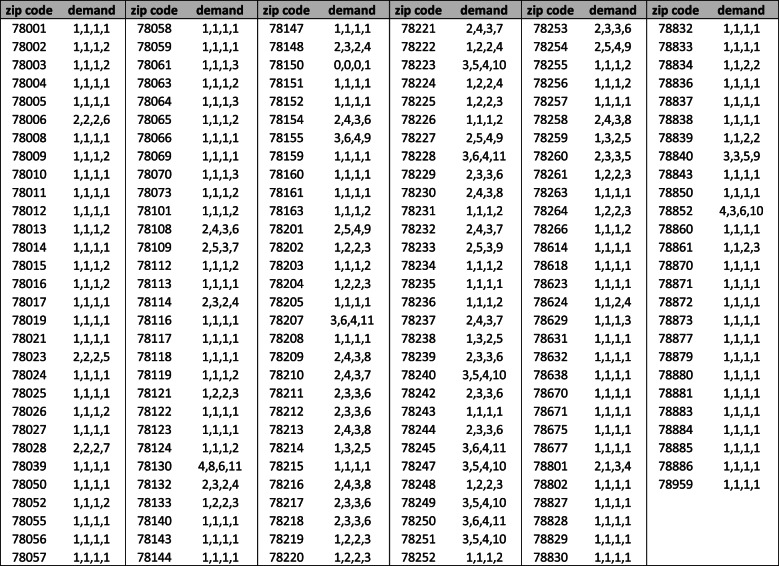
Combined (type $\mathcal l=1$ and $\mathcal l=2$) forecasted daily demand per scenario $\omega$
